# Tibial post fracture pain is reduced in kinin receptors deficient mice and blunted by kinin receptor antagonists

**DOI:** 10.1186/s12967-019-2095-9

**Published:** 2019-10-22

**Authors:** Vincent Minville, Lionel Mouledous, Acil Jaafar, Réjean Couture, Anne Brouchet, Bernard Frances, Ivan Tack, Jean-Pierre Girolami

**Affiliations:** 1 Department of Anesthesiology and Intensive Care, Toulouse University Hospital, Toulouse, France; 2 INSERM U 1048, I2MC, BP 84225, 31432 Toulouse Cedex, France; 3 Centre de Recherches sur la Cognition Animale, CNRS UMR 5169, Université P Sabatier, bat 4R3, 118 route de Narbonne, 31062 Toulouse Cedex, France; 4 CHU de Toulouse, Service d’Explorations physiologiques rénales, 31059 Toulouse cedex, France; 5 Department of Physiology, Medical School, University of Montreal, Montreal, QC H3C 3J7 Canada; 6 Department of Pathology, Centre Hospitalier Universitaire de Toulouse, Toulouse, France; 7 Department of Anesthesiology and Intensive Care, Rangueil University Hospital, Avenue, Jean Poulhès, Toulouse, France

**Keywords:** Pain, Bradykinin, B1 receptor, B2 receptor, Fracture, Orthopedic, Analgesia

## Abstract

**Background:**

Tibial fracture is associated with inflammatory reaction leading to severe pain syndrome. Bradykinin receptor activation is involved in inflammatory reactions, but has never been investigated in fracture pain.

**Methods:**

This study aims at defining the role of B1 and B2-kinin receptors (B1R and B2R) in a closed tibial fracture pain model by using knockout mice for B1R (B1KO) or B2R (B2KO) and wild-type (WT) mice treated with antagonists for B1R (SSR 240612 and R954) and B2R (HOE140) or vehicle. A cyclooxygenase (COX) inhibitor (ketoprofen) and an antagonist (SB366791) of Transient Receptor Potential Vaniloid1 (TRPV1) were also investigated since these pathways are associated with BK-induced pain in other models. The impact on mechanical and thermal hyperalgesia and locomotion was assessed by behavior tests. Gene expression of B1R and B2R and spinal cord expression of c-Fos were measured by RT-PCR and immunohistochemistry, respectively.

**Results:**

B1KO and B2KO mice demonstrated a reduction in post-fracture pain sensitivity compared to WT mice that was associated with decreased c-Fos expression in the ipsilateral spinal dorsal horn in B2KO. B1R and B2R mRNA and protein levels were markedly enhanced at the fracture site. B1R and B2R antagonists and inhibition of COX and TRPV1 pathways reduced pain in WT. However, the analgesic effect of the COX-1/COX-2 inhibitor disappeared in B1KO and B2KO. In contrast, the analgesic effect of the TRPV1 antagonist persisted after gene deletion of either receptor.

**Conclusions:**

It is suggested that B1R and B2R activation contributes significantly to tibial fracture pain through COX. Hence, B1R and B2R antagonists appear potential therapeutic agents to manage post fracture pain.

## Background

Tissue damage and inflammation lead to the activation of several inflammatory and pain systems among which is the kallikrein–kinin system. Kallikreins are proteolytic enzymes, which generate kinins, namely bradykinin (BK) and related peptides (Lys-BK) including their active metabolites from kininogen substrates [1]. The nonapeptide BK is an initial mediator of inflammation that induces pain [2–5]. The actions of kinins are mediated through the activation of two G-protein coupled receptors, B1 and B2. It was first hypothesized that the B1 receptor (B1R) is only expressed as a result of tissue damage and inflammatory signals whereas the B2 receptor (B2R) is constitutively expressed [1]. Consistently, B1R and B2R knockout mice showed hypoalgesia against inflammatory pain stimuli [6, 7] confirming the role of kinin receptors in pain signaling pathways in response to tissue damage and inflammation. The involvement of kinin receptors in inflammatory and neuropathic pain processes is now well recognized and extensively reviewed [5, 7]. Signaling relaying kinin receptors involves prostanoids and leucotrienes synthesis and sensitization of ion channels, especially the Transient Receptor Potential Vanilloid 1 (TRPV1) and Transient Receptor Potential cation channel subfamily A or M8 (TRPA1 and TRPM8, respectively) [5, 8, 9].

Several animal pain models exist to assess the efficacy of drugs or to study the physiopathology of pain [5]. Recently, we have described a new model of fracture pain in mice [10]. Tibial fracture models were first developed in rats [11] and have been used to investigate several systems involved in the so-called Complex Regional Pain Syndrome (CRPS) [12]. In human CRPS formerly known as Sudeck’s dystrophy is a disabling pain syndrome occurring after fractures or lesions of the peripheral or Central Nervous System (CNS), which is associated with mechanical and thermal hyperalgesia and allodynia at the level of the skin. Several physiopathological concepts involving various mechanisms have been proposed but no clear therapeutic strategy has yet emerged thereby justifying further investigations on animal models. It was first demonstrated that substance P signaling contributes to the vascular and nociceptive abnormalities in a tibial fracture rat model of CRPS [13]. From these studies in rats, it is known that anti-nerve growth factor (NGF) antibodies reduced some but not all signs characteristic of CRPS, illustrating the complexity of CRPS pathogenesis and NGF signaling [14, 15]. Recently, the same authors reported that IL-1beta contributes to chronic regional nociceptive sensitization after fracture, possibly by stimulating NGF overexpression in keratinocytes [16–20]. Collectively, these studies support the involvement of inflammatory cytokines, including TNF-alpha [14, 15] in the nociceptive sensitization after fracture. Moreover, it has been proposed that the upregulation of cytokine production depends on the activation of multi-protein complexes termed inflammasomes, which could be involved in the processing and activation of pro-inflammatory cytokines [16–20]. However, most of these investigations have focused on the chronic signaling pathways leading to post-traumatic chronic inflammation and pain mechanisms rather than on the potential upstream targets. In that respect, the kallikrein/kinin system could be recruited very early during the acute post-fracture pain, a possibility never investigated. For instance, pro-inflammatory cytokines are known to induce and upregulate B1R through the activation of the transcriptional nuclear factor NF-kappaB in several experimental models [21–24].

Therefore, the aim of this study was to assess the role of B1R and B2R in a model of post fracture pain in kinin receptor deficient mice. Moreover, we investigated the potential analgesic effect of B1R and B2R antagonists and of cyclooxygenase (COX) and TRPV1 receptor inhibitors in the mouse fracture model. The results identify B1R and B2R as early therapeutic targets in this clinically relevant acute fracture pain model.

## Materials and methods

### Animals

Animal experimentation was authorized by the French Direction of Veterinary Service to J.P.G., L.M., and B.F. The study was approved by The French Animal Care and Use Committee (Comité régional d’éthique pour l’expérimentation animale Midi Pyrénées, ref: MP 02/02/02/06). Experiments were performed in accordance with the guidelines for the care and use of laboratory animals and the ethical guidelines for investigations of experimental pain in conscious animals [25], and the European Communities Council Directive of November 24 1986 (86/609/EEC). Maximal efforts have been made to minimize the number of animals used and their suffering. Experiments were conducted using male wild-type (WT) C57 BL/6 mice (Janvier Lab., Le Genest St Isle France) and mice deficient for the B1 (B1KO) and B2 (B2KO) kinin receptors (C57 BL/6 background). The B2KO colony was established in our laboratory from genitors kindly donated by Dr. F. Hess (Merck Research Laboratory, Princetown, USA), whereas B1KO mice were provided by Professor M. Bader (Berlin, Germany). The animals were housed in isolator cages with solid floor covered with 3 cm of soft bedding and they had free access to standard chow diet and tap water. Animals were maintained on a 12 h light–dark cycle. The following inhibitors were used: B2R antagonist (Icatibant also named HOE 140) from Sanofi Aventis (Frankfurt Germany), B1R antagonist (SSR 240612), known to cross the blood–brain barrier (BBB), from Sanofi Aventis (Frankfurt Germany), the B1R antagonist R954, which does not pass the BBB was generously provided by Pr F. Gobeil Jr (Sherbrooke, Canada). The COX-1/COX-2 inhibitor ketoprofen was obtained from Sanofi Aventis (Montpellier, France) and the TRPV1 antagonist SB 366791 was purchased from Tocris Bioscience (Minneapolis, USA).

### Surgery

Unilateral closed tibial fracture was performed under 2%-sevoflurane (Abbott, Suresnes France) anesthesia as previously described [10]. Briefly, after antiseptic preparation of the right paw with povidone iodine, a closed fracture was produced using a specially designed fracture apparatus (blunt guillotine). Before fracture, an intramedullary pinning was performed under sterile conditions; a hole was made above the tibial tuberosity percutaneously using a 27-gauge needle (BD, Drogheda, Ireland). Then the needle was directed into the medullary canal. By rotating the needle, the canal was reamed to 5 mm up to the ankle joint. The end of the needle was cut as short as possible so that the skin could roll over and cover it. No suture was used. Then, the mouse was placed with the leg on the anvil so that the blunt guillotine lined up with the proximal third of the tibia. The 300 g weight was dropped from a height of 9–10 cm fracturing the tibia shaft. Closed fracture was confirmed post mortem by examining whether the nail is intra medullary or not. Only mice with intra-medullary inserted nail were included in the study.

### Behavioral assay

Three tests were used to assess pain behavior: (i) Mechanical nociception assessed by the withdrawal response to von Frey filament application, (ii) Thermal nociception assessed by the withdrawal response to thermal stimulus (hot plate test), and (iii) Subjective pain determined using a pain rating scale as described by Attal et al. [26].

### Mechanical nociception

Unrestrained mice were placed beneath a clear plastic chamber on an elevated mesh floor and allowed to acclimate. Withdrawal responses to mechanical stimulation were determined using calibrated von Frey filaments (from 0.008 to 8 g) applied from underneath the cage through openings in the plastic mesh floor against the hind paw plantar skin at approximately middle of the fractured leg. The filament was pushed until it slightly bowed and then it was maintained in that position for 6 s. Each von Frey filament was applied once starting with 0.008 g and continuing with higher force filaments until a withdrawal response was reached, which was considered as a positive response. The test was repeated 3 times. The lowest force from the three tests producing a response was considered the withdrawal threshold.

### Thermal nociception

Thermal nociception was measured by a modified hot-plate test [27]. The time that a mouse leaves its hind paw on a hot plate at 52 °C reflects thermal nociception (thermal latency). The paw was removed from the plate after a maximal time of 12 s by the investigator to avoid thermal injury and thermal hyperalgesia. This test was repeated three times on each hind paw for each mouse.

### Subjective pain scale

A subjective pain rating scale (0–5) modified from that previously described [28] was used to quantify pain, where: 0 is normal, 1 is curling of the toes, 2 is eversion of the paw, 3 is partial weight bearing, 4 is non-weight bearing and guarding, and 5 is avoidance with any contact with the hind limb.

### Locomotors activity: Actimeter

The device (Apelex^®^) has five Plexiglas boxes, identical dimensions of 25 cm (length) × 21.5 cm (width) × 9.5 cm (height). Two beams in each box to address the movement of mouse. All the boxes are cut from light, sound, and the external environment. Locomotors activity was measured using counters within the number of times mice crossed beams. The tests lasted 30 min and were made before and after the surgery.

### Experimental groups

To investigate the impact of B1R and B2R genetic deletion on pain behavior, three groups of mice were subjected to closed fracture as described above: Group 1 (wild-type WT mice, n = 9); Group 2 (B2KO, n = 9); Group 3 (B1KO, n = 9). All mice were tested before surgery (baseline), 2 h, 4 h and 6 h after surgery, and then once daily during the first 7 postoperative days, and each week during the 4 weeks following the surgery. Pain behavior tests included mechanical stimulation, hot plate test and pain rating.

To assess the possible involvement of the COX pathway, the effect of the COX-1/COX-2 inhibitor ketoprofen (50 mg/kg, s.c.), was tested in three additional groups of mice (n = 6) after production of a tibial closed fracture: Groups 4, 5 and 6 were WT, B1KO and B2KO treated with ketoprofen. In comparison, Groups 1, 2 and 3 were WT, B1KO and B2KO receiving a saline vehicle only. Pain behavior testing (mechanical stimulation, hot plate test, and pain rating) was performed before the surgery (T0 = baseline) and each 30 min up to 240 min after administration of ketoprofen or saline (administered at T0, just after basal assessment). The effect of ketoprofen administration was also assessed on behavioral testing on postoperative day 1 and 2 using the same procedure. Experiments were conducted following a double blind protocol.

To investigate the effect of pharmacological inhibition of B1R and B2R, WT mice subjected to a closed fracture were randomly assigned in four groups as follows: Group 1 (n = 6) received the saline vehicle subcutaneously postoperatively, Group 2 (n = 6) received the B2R antagonist HOE 140 (250 µg/kg subcutaneously) postoperatively, Group 3 (n = 6) received the B1R antagonist SSR 240612 (10 mg/kg/oral) postoperatively and Group 4 (n = 6) received R954 (1 mg/kg subcutaneously) postoperatively. The doses of B1R and B2R antagonists used in this study were based on previous experiments of our groups [24, 29]. SB-366791 [*N*-(3-methoxyphenyl)-4-chlorocinnamide], a potent and highly selective TRPV1 antagonist, was administered intraperitoneally at a dose of 1 mg/kg dissolved in 1% DMSO. Inhibitors (receptor antagonists, COX inhibitors) or saline or 1% DMSO vehicle were administered at T0. Responses to mechanical, heat stimuli, and pain rating scale were determined every 30 min after T0 for 240 min, then 300, 480 min and 24 h after inhibitor administration. Experiments were conducted following a double blind protocol.

### Kinin receptors mRNA expression using real time PCR (RT-PCR)

Two days after surgery, mice (n = 6) were sacrificed (anesthesia overdose using pentobarbital) and both the fractured and contralateral non-fractured tibia were dissected out. The RNA from both tibias was extracted from control mice, i.e. WT, B2KO and B1KO mice without fracture using kit RNAeasy kit (QIAGEN, S.A., Courtaboeuf, France) according to the manufacturer’s instructions. Reverse transcription was performed using specific primer to mRNA of kinin B2R and B1R (Eurogentec, Belgium). RT-PCR was performed in triplicate. Amplification and detection were performed using an ABI PRISM 7000 Sequence Detector system (Applied Biosystems, USA). 18S cDNA was amplified in parallel to normalize the expression levels of B1R and B2R mRNAs.

### c-Fos spinal cord immunohistochemistry

c-Fos immunohistochemistry was performed as previously described [28]. Mice were deeply anesthetized with pentobarbital 2 h after surgery and perfused transcardially with saline followed by 4% paraformaldehyde (PFA) using a peristaltic pump. Lumbar spinal cords were dissected out and postfixed overnight at 4 °C in 4% PFA, then transferred into 30% (w/v) sucrose buffer for 48 h at 4 °C before freezing in isopentane. 20 µm thick coronal sections were cut at the L3–L5 levels using a Microm Microtech cryostat (HM 500 M, Francheville, France). Sections were incubated with 0.3% H_2_O_2_ in PBS for 30 min at room temperature before exposure for 2 h to a PBS blocking solution containing 0.25% Triton X-100 and 1.5% normal goat serum (Vector Laboratories, Burlingame, CA, USA). Sections were then incubated with rabbit anti-c-Fos antibody (Santa Cruz Biotechnology, Santa Cruz, CA, USA) diluted 1:20,000 in the same buffer for 48 h at 4 °C followed by biotinylated goat anti-rabbit antibody (Vector Laboratories) diluted 1:200 for 2 h at room temperature. After washing, sections were placed in horseradish peroxidase avidin-biotin complex (Vectastain ABC kit, Vector Laboratories) diluted in PBS 0.5% with Triton X-100 for 1 h at room temperature. Sections were finally stained in 3,3’-diaminobenzidine substrate kit (Vectastain DAB kit, Vector Laboratories) for 6 min and mounted on gelatin-coated slides. Sections were observed under a Nikon Eclipse 80i microscope (Champigny sur Marne, France) and pictures of the area of interest were taken using a Nikon Digital Sight DS-5M-L1 camera and software under the 20× objective. Total number of c-Fos positive nuclei was quantified in superficial layers I and II of the L3–L5 spinal cord (6 mice per condition, 3 sections per mouse) on thresholded images using the Image J software (NIH, USA).

### Western blot analysis

B1R and B2R proteins were separated by SDS PAGE and analyzed by Western Blotting as previously described [23, 30]. Proteins were extracted using a lysis buffer containing 10% glycerol, 20 mM Tris, 140 mM NaCl, 3 mM EDTA, protease and phosphatase inhibitor cocktails (Roche, Germany), and 1% Triton X-100. In agreement with the manufacturer (BD-Biosciences, USA) technical data sheet B2R receptor was revealed as a double band which was absent in the B2R deficient mice. The B1R polyclonal antibody was previously characterized in the laboratory [23].

### Histology and immunohistochemistry

Tissue samples were fixed in 10% formalin and embedded in paraffin after fast decalcification (Decalcifier, Thermo shandon TBD1). Sections of 4–5 microns were made and stained with Haematoxylin Eosin for morphological study. Immunohistochemical studies were performed on histological sections from the same block, with the anti-CD 68 antibody (Dako), which recognizes the monocyte/macrophage (1/100, 30 min), anti CD-34 (Immunotech), which marks the vessels (1/100, 30 min) and anti CD 45 (Dako), which indicates leukocytes (1/100, 30 min). The revelation was made by the avidin–biotin-peroxidase technique (LSAB kit).

### Collagen deposition

The presence of collagen deposition was demonstrated by specific Syrius Red stainings, briefly, the sections were deparaffinized (3 toluene baths, 5 min) and then rehydrated by three successive baths of ethanol (100°, 95° and 50°, 5 min) and a water bath (5 min). Sections were incubated 30 min in a solution of Sirius Red (0.1% F3B saturated picric acid) before being rinsed with water 5 min. The slides were observed under an optical microscope (Eclipse E400, Nikon) at a magnification ×2. Photographs (Microfire, Optronics) were analyzed using the 3.0 software MorphoExpert to allow quantification of the surface measured by the collagen relative to the total area of tissue fibrosis.

### Statistical analysis

Since values for behavior were not normally distributed, they were subjected to nonparametric statistical analysis. To assess whether the withdrawal responses changed over time, Friedman’s test was used. When Friedman’s test was found significant (P < 0.05), pairwise comparisons were made using Wilcoxon’s signed rank test. Time point comparisons between groups were made using a nonparametric Kruskal–Wallis. When Kruskal–Wallis test was significant (P < 0.05), pairwise comparisons were made using Mann–Whitney U test.

The c-Fos results were expressed as mean ± SEM of the number of positive nuclei/section. Statistical analyses were performed using GraphPad Prism software (GraphPad, San Diego, CA, USA). One-way ANOVA were followed by *post*-*hoc* multiple comparisons using Bonferroni’s test.

## Results

Throughout the experimental period, all mice remained well-groomed and maintained normal food and water intake. No change in body weight, no signs of spontaneous pain behavior, such as licking, biting, and flinching, were noticed after the surgery.

### Fracture pain is blunted in the absence of kinin receptors

**Fig. 1:**
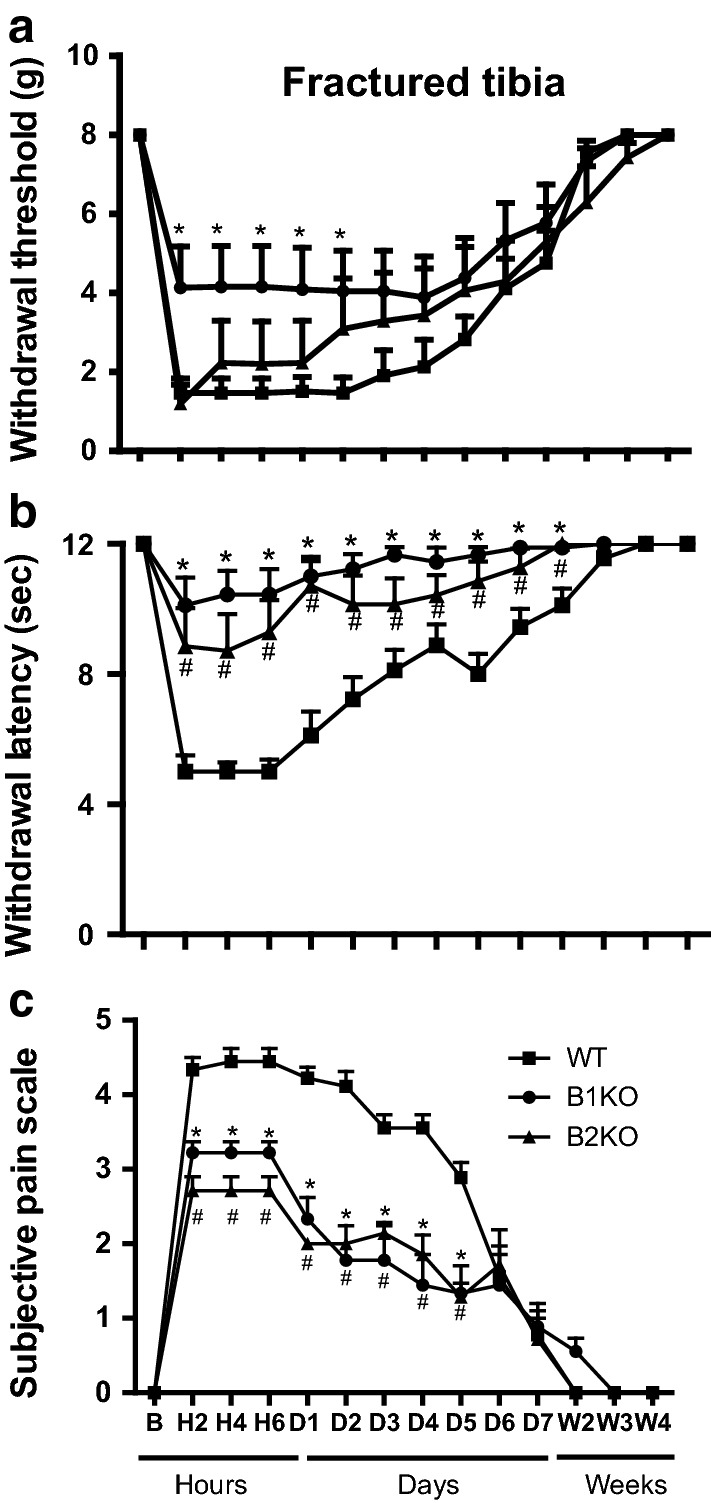
Mechanical (**a**), thermal (**b**) hyperalgesia and subjective pain (**c**) caused by fractured tibia are reduced in B1 and B2 receptor knockout (B1KO, B2KO) compared to wild-type (WT) mice. After baseline testing, male adult mice were subjected to closed tibial fracture and tested for paw withdrawal threshold to evaluate mechanical sensitivity (**a**) or paw withdrawal latency to evaluate thermal sensitivity (**b**). Subjective pain (**c**) was expressed by a rating scale from 1 to 5 as described in “Materials and methods” section. The contralateral side was also tested at each time point for each group of mice: the mechanical thresholds were 8 g (cut off), the heat latencies were 12 s (cut off), and the subjective pain scores were zero in all groups at all time points. Each bar represents mean ± SEM of 9 mice per group. Data were subjected to Friedman’s test followed by Wilcoxon’s signed rank test. *P < 0.05 when comparing B1KO to WT. ^#^*P* < 0.05 when comparing B2K0 to WT

**Fig. 2:**
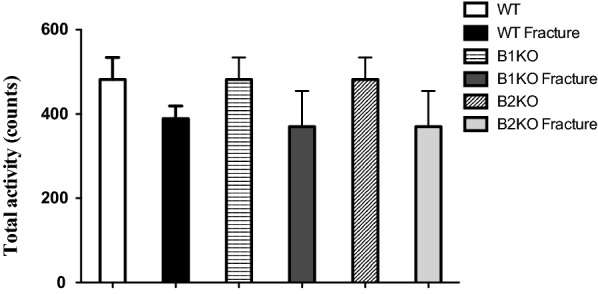
Locomotors activity. Overall locomotors activity was recorded by an actimeter preoperatively and postoperatively. Locomotors function was not different in mice (WT, B1KO and B2KO) before and after the surgery. Data are expressed as means ± SEM

Baseline values for pain behavior parameters were not significantly different between groups before fracture induction (Fig. 1). Similarly, no behavioral modification occurred in the non-fractured tibia mice (data not shown). After fracture, behavioral pain measurements were significantly but differently reduced both in B1KO and B2KO mice when compared to WT mice (Fig. 1). Maximal pain was observed in WT animals. In B1KO mice, both mechanical and thermal sensitivity were significantly and persistently reduced from 2 h up to 7 days post fracture. In B2KO mice no difference was observed in mechanical sensitivity whereas thermal sensitivity was reduced to a similar level as that observed in B1KO. The subjective pain scale was significantly lower both in B1KO and B2KO mice when compared to WT mice from 2 h to 5 days. All mice recovered to control values 2 weeks after fracture and no rebound in pain sensitivity was observed up to 4 weeks post-fracture. Concerning the locomotors function of the mice, no difference was found before the fracture or after the fracture concerning WT, B1KO and B2KO mice (Fig. 2).

### Effect of absence of kinin receptors on c-Fos induction during tibial fracture

**Fig. 3:**
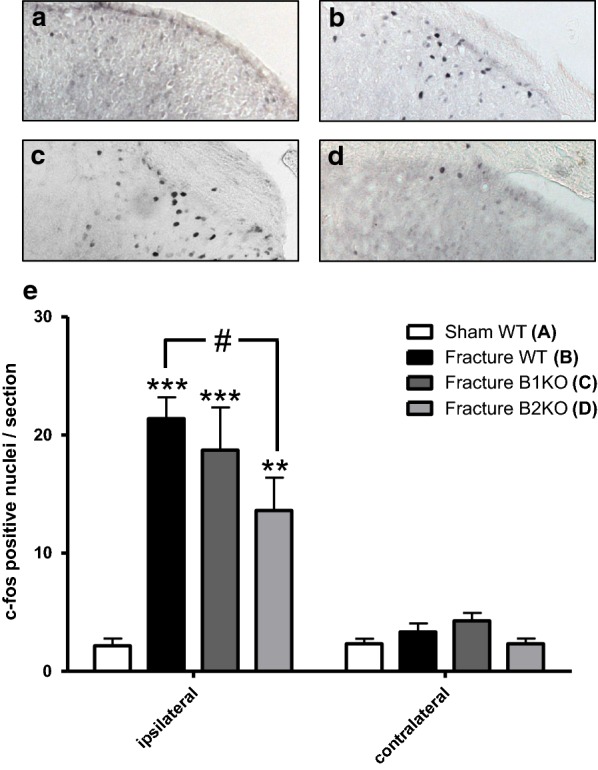
Fracture-induced c-Fos expression is reduced in the superficial layers of the spinal cord dorsal horn in B2 receptor knockout mice (B2KO). c-Fos expression was assessed by immunohistochemistry at the L3–L5 level 120 min after fracture induction. **a**–**d** Are photomicrographs of c-Fos labeling from representative sections obtained from sham wild-type (WT) (**a**), fractured WT (**b**), fractured B1 receptor knockout mice B1KO (**c**) and fractured B2KO (**d**) mice, ×20 objective. Images are zoomed on layers 1 and 2 were most of the c-Fos labelling is observed. In (**e**), bar graph illustrating the mean ± SEM of the number of c-Fos positive nuclei/section from six animals in each group. **P < 0.01, ***P < 0.001 compared to ipsilateral sham WT. One-way ANOVA followed by Bonferroni’s post-hoc test. ^#^P < 0.05 compared to ipsilateral fractured WT

c-Fos immunohistochemistry was performed to study the initial response to fracture in the dorsal horn of the spinal cord in the three mouse strains. As shown in Fig. 3, 2 h after tibial fracture there was a significant increase in c-Fos immunoreactivity in the superficial layers of the ipsilateral L3–L5 dorsal horn of WT mice compared to sham animals and to the contralateral side. The number of c-Fos positive nuclei was similar to WT in fractured B1KO mice whereas it was significantly reduced in B2KO fractured mice.

### Kinin receptors mRNA and protein expression

**Fig. 4:**
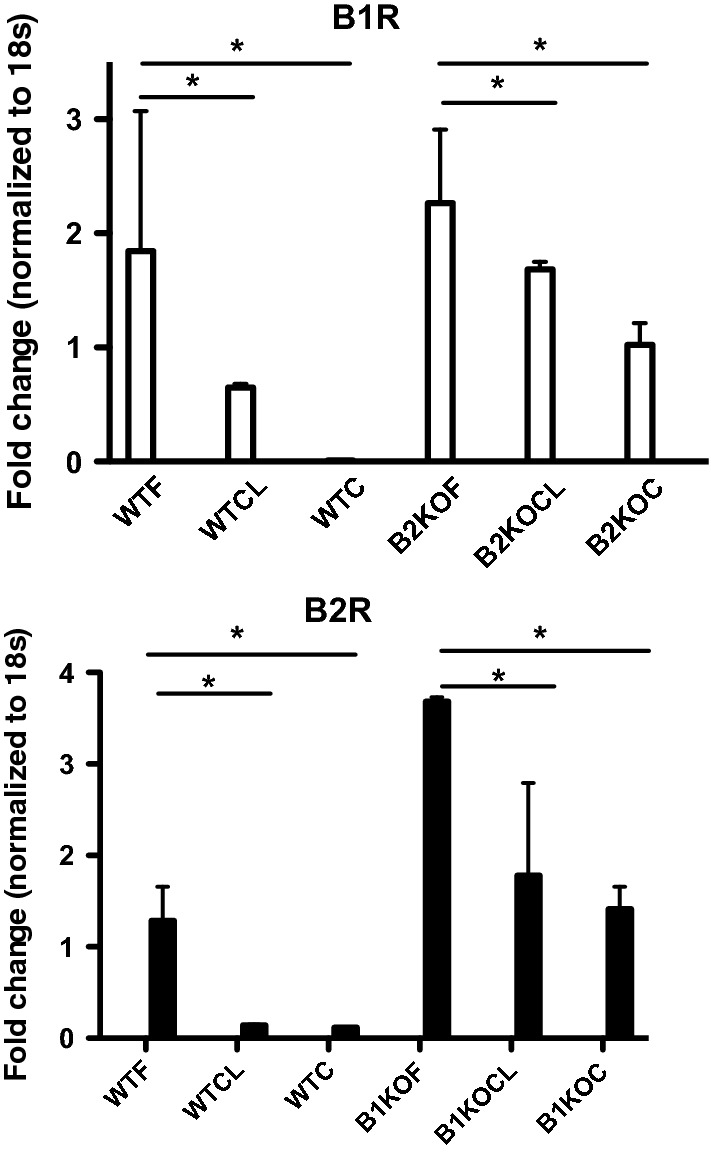
B1 and B2 receptor mRNA expression is maximal at the site of fracture. Two days after fracture, mice were sacrificed and RNA was extracted from the fractured (F) and contralateral (CL) non-fractured limbs. RNA was also extracted from control (C) mice without fracture (i.e. WTC or B2KOC, B1KOC mice). Each bar represents mean ± SEM of at least 6 mice per group, Data were subjected to Wilcoxon signed rank test. *P < 0.05 compared to ipsilateral fractured WT

In WT mice, B1R and B2R mRNA levels in the tibia were significantly increased at 2 days after fracture when compared to the non-fractured contralateral side and control mice (Fig. 4). In B1KO and B2KO mice, basal expression of the remaining receptor (B2R and B1R, respectively) was significantly higher than in WT. In addition, as in WT mice, B2R (in B1KO) and B1R (in B2KO) mRNA levels were significantly increased in the fractured side when compared to both the non-fractured side and control mice.

**Fig. 5:**
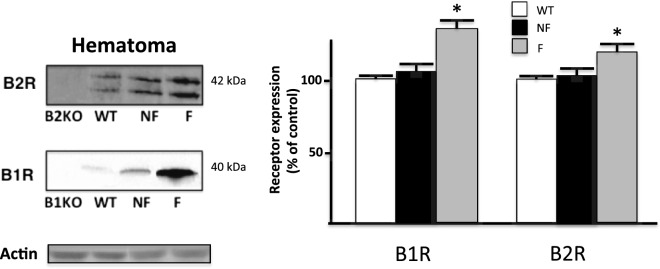
Bradykinin receptor and protein expression. B1-kinin receptor (B1R) and B2-kinin receptor (B2R) expression was analyzed by Western blotting. Protein extracts were obtained from wild control type mice (WT) and from mice with tibial fracture, samples from the fractured side (F) and contralateral non-fractured (NF) side were analyzed. Proteins extracts from hematoma were compared. Tissue sample from B1R and B2R deficient mice were used to check the antibody specificity. The specific of both B1R and B2R antibodies was attested by the absence of protein obtained from B1R and B2R KO mice. Qualitative differences in protein expression of B1R and B2R were shown and were further quantified by densitometry. Results shown in bar graphs are presented as arbitrary units by comparison to expression in WT taken as control. *P < 0.01 when compared to expression in WT

As shown in Fig. 5, an increase in B1R and B2R was evidenced in the extracts obtained from tissue surrounding the site of fracture (hematoma), thus corroborating mRNA data. However, no change in B1R or B2R protein expression could be detected by Western blot in fractured bone, paw skin extracts, and in total extracts of L3–L5 spinal cord of injured mice (data not shown).

### Pharmacological inhibition of kinin receptors reduced fracture pain

**Fig. 6:**
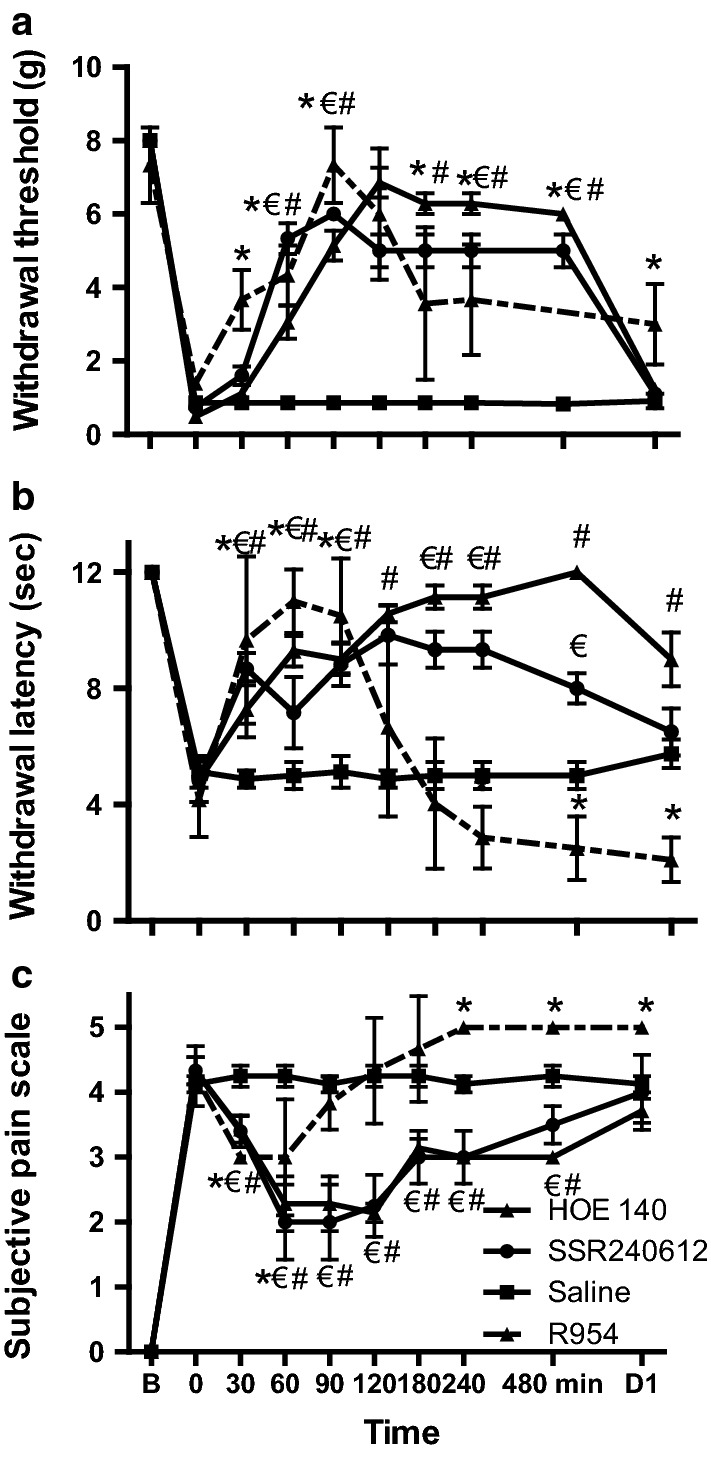
B1 and B2 receptor antagonists reduce mechanical (**a**), thermal (**b**) sensitivities and subjective pain (**c**) on the fractured tibia side in wild-type (WT) mice. Experiments were conducted as described in Fig. 1. At time 0, the B2 receptor antagonist Icatibant (HOE 140) was given as a single subcutaneous injection (250 µg/kg). B1 receptor antagonists were given either as gavage using SSR240612 (10 mg/kg body weight) or as a single subcutaneous injection of R954 (10 mg/kg body weight). Behaviors were recorded for 8 h and then once the subsequent day 24 h after drug administration. Each bar represents mean ± SEM of 6 mice per group. B: baseline. Data were subjected to Friedman’s test followed by Wilcoxon’ signed rank test. *, ^#^ and ^€^*P* < 0.05 compared with saline-treated WT mice

Before induction of fracture, different groups of WT mice were treated with either a B2R antagonist or two distinct B1R antagonists. Withdrawal threshold to mechanical stimulation (Fig. 6a) and withdrawal latency to thermal stimulation (Fig. 6b) increased transiently after administration of B2R or B1R antagonists. Blockade of B1R and B2R with SSR240612 and HOE 140 induced a significant reduction of fracture pain, which lasted up to 4 h. The subjective pain scale (Fig. 6c) was also significantly reduced for up to 4 h following SSR240612 and HOE 140 administration. Blockade of B1R with the R954 antagonist was also efficient but the inhibition of pain behavior was more transient with a maximum efficacy peaking at around 1 h post-administration. Surprisingly, after this initial analgesic phase, R954 increased significantly pain sensitivity above saline values both on thermal pain (1 day post-injection) and subjective pain (between 4 h and 1 day after injection).

### Inhibition of COX-1/COX-2 had no effect on fracture pain in the absence of kinin receptors

**Fig. 7:**
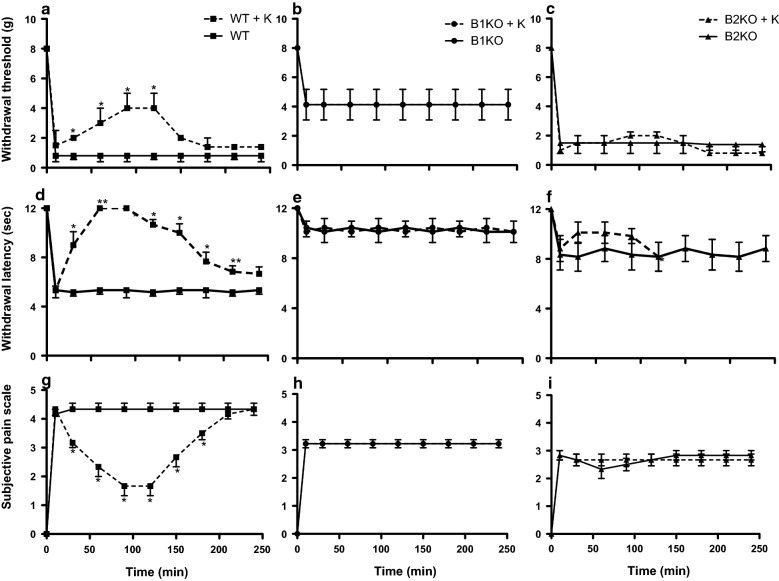
COX inhibition with ketoprofen (K) reduces pain sensitivities in wild-type (WT) mice but has no effect in B1 and B2 receptor knockout (B1KO, B2KO) mice. WT, B1KO and B2KO were subjected to tibial fracture and received a single injection of K (50 mg/kg body weight) at time 0. Behaviors were recorded during 4 h. Each bar represents mean ± SEM of 6 mice per group. Data were subjected to Friedman’s test followed by Wilcoxon signed rank test. *P < 0.05 compared with fractured untreated WT

In WT mice, withdrawal threshold to mechanical stimulation (Fig. 7a) and withdrawal latency to thermal stimulation (Fig. 7d) were increased transiently after ketoprofen administration compared to saline and the effect lasted between 120 and 240 min depending on the test. Subjective pain was also transiently reduced (Fig. 7g). In contrast, ketoprofen had no effect on the residual mechanical and thermal sensitivity observed in B1KO (Fig. 7b, e, h) and B2KO (Fig. 7c, f, i) mice.

### Inhibition of TRPV1 still reduced fracture pain in the absence of kinin receptors

**Fig. 8:**
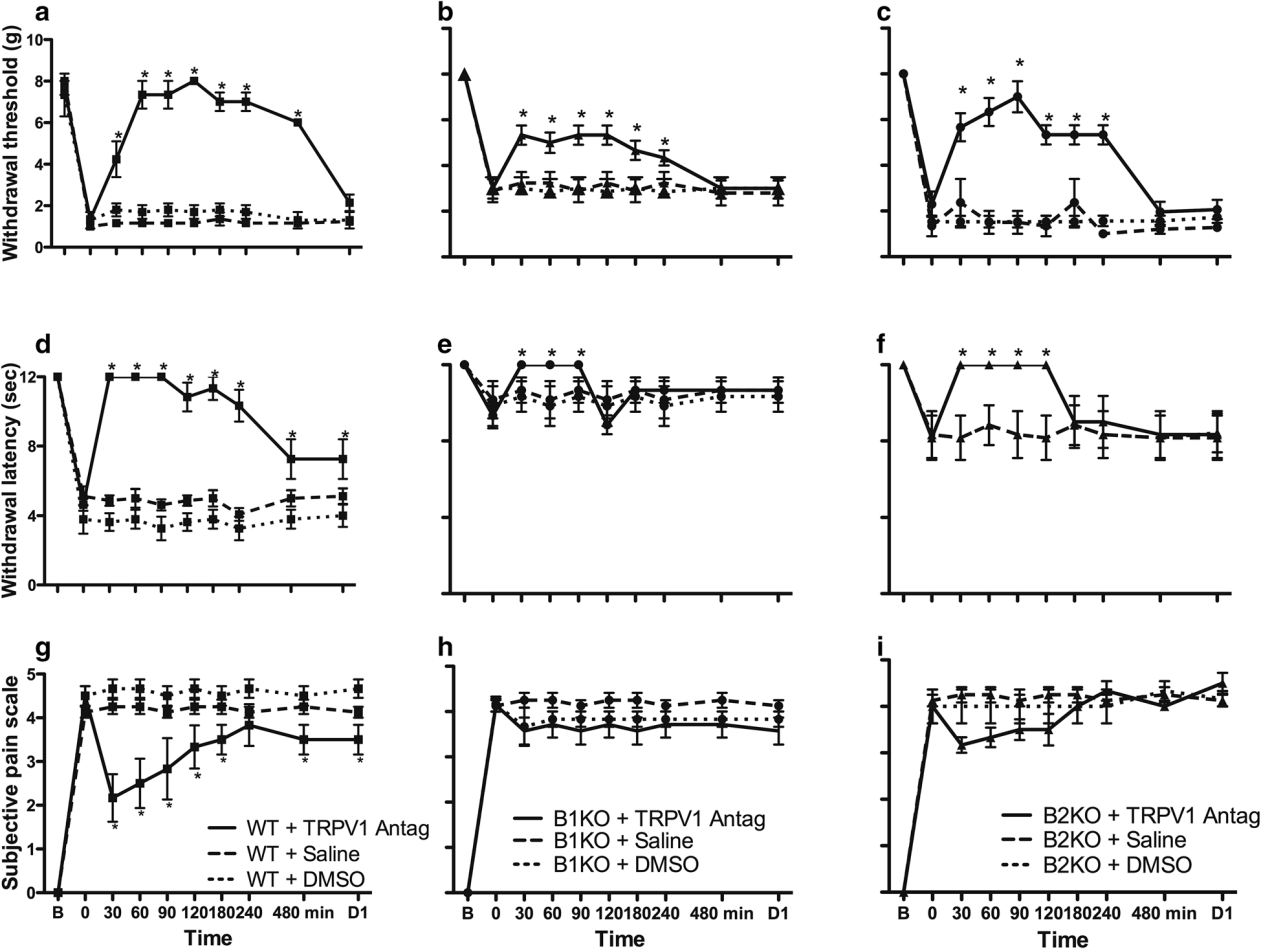
TRPV1 inhibition reduces pain sensitivities in wild-type (WT) and B1 and B2 receptor knockout (B1KO, B2KO) mice. WT, B1KO and B2KO mice were subjected to tibial fracture and received a single injection of SB 366791 (1 mg/kg) at time 0. Behaviors were recorded during 8 h and the subsequent day. Each bar represents mean ± SEM of 6 mice per group. Data were subjected to Friedman’s test followed by Wilcoxon signed rank test. *P < 0.05 compared with fractured untreated, i.e. WT, B1KO and B2KO + saline or + DMSO respectively

In WT mice, withdrawal threshold to mechanical stimulation (Fig. 8a) and withdrawal latency to thermal stimulation (Fig. 8d) were significantly but transiently (up to 8 h) increased after treatment with the TRPV1 antagonist (SB366791) in comparison with saline. Subjective pain was also transiently reduced (Fig. 8g). Importantly, the TRPV1 antagonist still reduced significantly mechanical and thermal sensitivity in B1KO (Fig. 8b, e) and B2KO (Fig. 8c, f) mice.

### Histology and immunohistochemistry

**Fig. 9:**
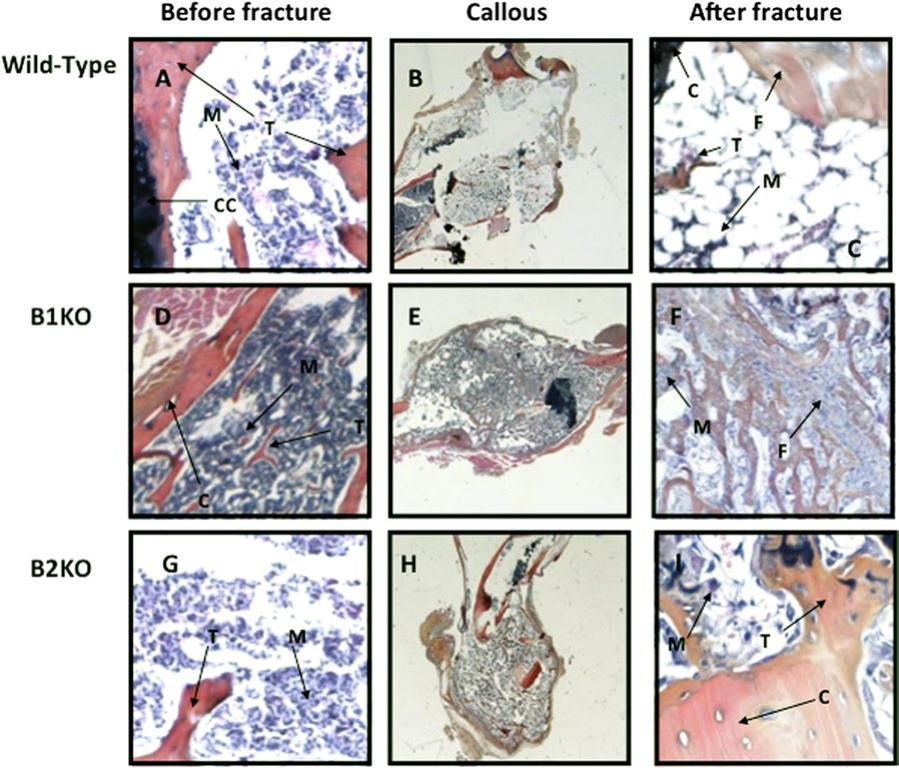
Histological analysis of non-fractured and fractured zones in all groups 4 weeks after the trauma. Wild-type mice (**a**–**c**), B1 receptor knockout (B1KO) (**d**–**f**) and B2 receptor knockout (B2KO) (**g**–**i**). There is no visible morphological difference between the B1KO and/or B2KO when compared to WT. *CC* Cartilage conjugation, *C* cortical, *T* trabecular bone, *M* bone marrow, *F* fibrosis

There is no visible morphological difference in B2KO and B1KO mice when compared to wild type mice before fracture (Fig. 9a, d and g). Four weeks after the fracture, we note the presence of fibrosis, which shows no significant morphological differences between B1KO and B2KO when compared to wild-type mice (Fig. 9b, e and h).

There was no difference between groups with respect to the expression of markers of vessels (CD34) or leukocyte (CD 45) (data not shown). However, an increase in the expression of osteoclast marker (CD 68) was found in all groups after the fracture when compared to its expression before the fracture. There was no significant difference between groups regarding the expression of CD 68 (data not shown).

#### Collagen deposition

An increased expression of collagen was found in the different groups after the fracture, no significant difference between groups was found (data not shown).

## Discussion

In the recent years, knowledge of the signaling pathways involved in chronic post-fracture pain has tremendously improved. The involvement of nerve growth factor and inflammatory cytokines and mediators has been carefully described using closed fracture model in rats [31]. Because of the recent availability of genetically modified mice, we have developed a fracture pain model in mice [10] which opens new possibilities to investigate physiopathological mechanisms [32]. In the present study, we describe for the first time that the absence of B1R or B2R reduces acute post-fracture pain. The data are consistent with the role of B1R and B2R in pain sensitization in inflammatory models. To confirm the involvement of B1R and B2R in acute fracture pain, we also demonstrated that pharmacological blockade with specific B1R or B2R antagonists significantly reduces post-fracture pain. Moreover, the pain mediated by these receptors is dependent on the COX signaling pathways as the COX-1/COX-2 inhibitor ketoprofen had no analgesic effect in the absence of B1R or B2R. By contrast, TRPV1, another important signaling pathway [5, 33] that conveys painful stimuli and is likely involved in post-fracture pain, appears to be partly independent of B1R and B2R activation.

Kinins are released in damaged tissues from circulating kininogen precursors by kallikreins and mediate rapid algogenic and pro-inflammatory effects. The involvement of B1R and B2R in inflammatory reaction and thereby in pain mechanism is documented in different pain models [5]. In knockout mice lacking both B1R and B2R, baseline nociceptive responses to heat and nociceptive responses to BK were abolished in acute acetic acid-induced visceral nociception while heat hypersensitivity in carrageenan induced paw inflammation was strongly reduced, although chronic inflammatory or nerve injury responses were unaltered [34]. In addition, the distinct role of each receptor can be explored by using specific antagonists and single receptor KO animals. Deletion of B2R, or treatment with a B2R antagonist, also abolished the nociceptive responses to intraplantar infusion of carrageenan indicating that B2R activation is an essential step in the initiation of the nociceptive response [7, 35]. Deletion of B2R and B2R receptor antagonist also prevented opioid-induced hyperalgesia confirming an important nociceptive role of the B2R [36]. In contrast, B1 agonists could not directly activate primary afferent neurons in a spinal nociceptive reflex preparation [37] or in dorsal root ganglions [38]. Therefore, it is likely that activation of the B2R rather that B1R is required to mediate the direct algogenic effect of BK in acute pain. This is consistent with the localization of B2R on sensory neurons and the ability of B2R antagonists to inhibit BK-induced nociception [40–41]. These data also suggested that although B1R are not involved in the initial algogenic effect, they contribute to inflammatory hyperalgesia secondary to acute pain.

Our data are consistent with these differential effects of B1R and B2R. First, the absence of B1R did not reduce c-Fos expression in the superficial layers of the spinal cord 2 h after the fracture whereas fracture-induced neuronal activation was reduced in B2KO. Considering the time course of this immediate early gene induction, this result suggests that B1R is not involved in the acute generation of fracture pain, possibly because B1R might not be located directly on sensory neurons but on surrounding tissue. Second, inflammation appears to be a prerequisite to ensure B1R induction and subsequent activation, as demonstrated here by RT-PCR and Western Blotting. These analyses showed that expression of B1R and B2R increased only in the tissue around the fracture. No protein over-expression could be detected either in the bone or in the L3–5 spinal cord segments or in the plantar skin of the paw. Taken together, this information does not support the expression of B1 receptors on sensory fibers but rather suggest that up regulation of these BK receptors occurs on infiltrating inflammatory cells (macrophages and leucocytes). Therefore the analgesic mechanism of B1R antagonists appears rather indirectly mediated likely by blunting the inflammatory response of infiltrating cells. The assumption of an indirect effect of the antagonists is also consistent with the efficiency of R954. R954 is a potent, selective and stable B1R antagonist with only peripheral action [42]. The distribution of R954 assessed by binding studies is restricted to peripheral tissues (liver, lung kidney) and hematological fluids with a very slow metabolizing rate over 24 h. Interestingly, this recent work also demonstrated that R954 is unable to cross the blood brain barrier and thus may not affect pain arising from central nervous structures. In the case of B2R, basal expression was detected in the skin and spinal cord. In addition to a possible action on inflammatory cells, the analgesic effect of HOE 140 could thus be exerted directly on sensory fibers.

Previous studies have demonstrated a role for B2R signaling in various animal pain models [5, 40]. In our study, we found that thermal hyperalgesia and subjective pain quotation were diminished in KOB2 fractured mice, but mechanical hyperalgesia was not affected. These results are consistent with prior studies that observed BK signaling contributed to heat but not mechanical hyperalgesia in animal pain models [43–46] and in humans [47]. In the present study, three behavioral tests indicate that acute treatment with either B1 or B2 antagonist reduced post-fracture pain in WT mice. As B1 receptor is not detectable in non-fractured WT mice and appeared specifically expressed in the fracture area, inhibition of B1R may be a potential therapeutic target for reducing fracture pain.

Interestingly, in the absence of B1R or B2R, inhibition of COX-1/COX-2 did not further reduce mechanical and thermal nociception. This observation is consistent with the induction of COX-2 following B1R and B2R activation as previously established [48, 49].

It has been proposed that TRPV1 activation is the final step in BK-induced heat sensitization [5, 50, 51]. The TRPV1 antagonist SB366791 did reduce post-fracture heat and mechanical hyperalgesia and subjective pain behavior in WT fractured mice, but this effect was not lost in the B1KO and B2KO mice indicating that TRPV1 involvement was not restricted to BK induced post-fractured hyperalgesia and pain behavior.

## Conclusion

This study utilized a genetic and pharmacologic approach to demonstrate that both kinin receptors (B1R and B2R) contribute to acute post-fracture pain through a downstream COX-1/COX-2 mechanism. These results suggest that blocking B1R and B2R signaling may be an effective therapeutic approach in the management of post-fracture pain.

## Data Availability

Please contact author for data requests

## Abbreviations

BK: bradykinin
B1R: B1 receptor
B2R: B2 receptor
B1KO: knockout mice for B1R
B2KO: knockout mice for B2R
WT: wild-type
COX: cyclooxygenase
TRPV1: Transient Receptor Potential Vaniloid1
CRPS: Complex Regional Pain Syndrome
CNS: Central Nervous System
NGF: nerve growth factor
RT-PCR: real time PCR
PFA: paraformaldehyde
